# Reduced prefrontal hemodynamic responses measured using near-infrared spectroscopy in adults with autism spectrum disorder

**DOI:** 10.3389/fpsyt.2024.1507890

**Published:** 2025-01-06

**Authors:** Kohei Kamikawa, Kazuhiko Yamamuro, Ryo Mizui, Natsuko Kashida, Rio Ishida, Takashi Okada, Nakao Iwata, Manabu Makinodan

**Affiliations:** ^1^ Department of Psychiatry, Nara Medical University School of Medicine, Kashihara, Japan; ^2^ Center for Health Control, Nara Medical University School of Medicine, Kashihara, Japan; ^3^ Department of Psychiatry, Fujita Health University School of Medicine, Toyoake, Japan; ^4^ Division of Transformative Psychiatry and Synergistic Research, International Center for Brain Science, Fujita Health University, Toyoake, Japan

**Keywords:** near-infrared spectroscopy, autism spectrum disorder, prefrontal cortex, hemodynamic responses, stroop color‑word task

## Abstract

**Aim:**

Functional neuroimaging studies have suggested that prefrontal cortex dysfunction occurs in individuals with autism spectrum disorder (ASD). Near-infrared spectroscopy (NIRS) is a noninvasive optical tool used to investigate oxygenation and hemodynamic responses in the cerebral cortex by measuring changes in oxygenated hemoglobin. Previous studies using NIRS have suggested that male children with ASD exhibit reduced hemodynamic responses in the dorsolateral prefrontal cortex; however, only a few studies examined this response in adults with ASD.

**Methods:**

We examined the characteristics of prefrontal hemodynamic responses in 114 adults with ASD and 84 typically developing controls. Relative concentrations of oxygenated hemoglobin were measured with frontal probes every 0.1 s during the Stroop color-word task, using 24-channel NIRS.

**Results:**

Our findings demonstrated that the oxygenated hemoglobin changes in the ASD group were significantly smaller than those in the control group at channels 19, 20, 23, and 24- located over the orbitofrontal cortex and frontal pole (*p <*0.05 for all three channels). The differences in oxygenated hemoglobin changes at Ch 20 were significantly correlated with the Autism-Spectrum Quotient Japanese version (AQ-J) total score and attention switching score, which is a symptom cluster of AQ-J (*p* = 0.043 and *p* = 0.009, respectively).

**Conclusion:**

Adults with ASD have reduced prefrontal hemodynamic responses as measured using near-infrared spectroscopy and the reduced activity of the frontal pole in particular is related to reduced attentional function.

## 1 Introduction

Autism spectrum disorder (ASD) is a neurodevelopmental disorder characterized by impaired social and communicative functions, restricted interests, and repetitive behaviors (1). A substantial body of research has demonstrated that individuals with Autism ASD exhibit impaired executive function (2–5). The executive function generally comprises three underlying components: response inhibition, updating working memory representations, and set shifting (6, 7). Previous research indicated that the prefrontal cortex (PFC) is strongly associated with response inhibition, a crucial component of executive function. For instance, patients with frontal lobe damage exhibit increased error rates and prolonged reaction times in the Stroop color-word task (8, 9). Functional neuroimaging studies, such as functional magnetic resonance imaging, have implicated multiple brain regions, including the anterior cingulate gyrus13 and dorsolateral PFC, in the accurate performance of this task (10).

Multichannel near-infrared spectroscopy (NIRS) can be utilized to non-invasively assess hemodynamic responses (cortical activity) in the cerebral cortex using near-infrared light (11, 12). NIRS quantifies alterations in oxygenated hemoglobin [oxy-Hb] and deoxygenated hemoglobin [deoxy-Hb] concentrations within the cerebral cortex. The hemodynamic responses in the cerebral cortex are reflected by local increases in [oxy-Hb] concentration and decrease in [deoxy-Hb] concentration (11, 13). Moreover, alterations in [oxy-Hb] concentrations of the cerebral cortex measured using NIRS demonstrate a correlation with the results obtained from positron emission tomography (PET) (14, 15).

NIRS has been utilized to evaluate cerebral function in subjects with diverse psychiatric disorders, including schizophrenia (16–19), bipolar disorder (19, 20), depression (16), obsessive-compulsive disorder (21–23), dementia (24, 25), post-traumatic stress disorder (26), Tourette’s disorder (27), attention-deficit/hyperactivity disorder (ADHD) (28–31), methamphetamine-induced psychosis (32, 33), and ASD (34, 35). NIRS is deemed to be advantageous for psychiatric research and clinical applications, particularly due to the following considerations. Firstly, NIRS can minimize the effects of motion artifacts, making it useful for experiments involving tasks that require movement, such as speech. Secondly, during measurement, the subject can maintain their typical seated posture, enabling data collection in an environment that closely approximates real-world conditions. Thirdly, it exhibits lower operational costs compared to alternative functional neuroimaging methodologies, demonstrates ease of implementation and utilization, and offers enhanced versatility. Furthermore, it exhibits high temporal resolution (less than 1 second) and possesses the capability to measure alterations in cerebral blood flow among patients with mental disorders in real time and longitudinally (16, 20, 26). These advantages of NIRS facilitate the conduct of neurofunctional imaging research under conditions where PET and functional magnetic resonance imaging (fMRI) measurements are challenging, and it is utilized in various fields beyond mental disorders.

Numerous review articles and meta-analyses have demonstrated that individuals with ASD, spanning childhood, adolescence, and adulthood, exhibit deficits in various aspects of executive function (36–40). The Stroop color-word task is one of the most frequently utilized executive function assessments to measure an individual’s capacity to inhibit a prepotent response in favor of an atypical response (41). This assessment evaluated both cognitive flexibility and inhibitory control (42). These cognitive functions are associated with the inferior frontal, dorsolateral prefrontal, and anterior cingulate cortices, and researchers have observed their activation during the administration of the Stroop color-word task number of correct answers (SCWT) (43, 44). Furthermore, multiple studies have documented suboptimal performance on the SCWT among various patient populations characterized by executive dysfunction (45).

Recent advancements in NIRS have facilitated the non-invasive examination of cerebral functions in various psychiatric disorders, including ASD. Previous investigations utilizing 16-channel (Ch) NIRS have demonstrated that no statistically significant difference was observed in hemodynamic responses within the prefrontal cortex during the Stroop color-word task between typically developing children and those diagnosed with ASD (46, 47). Conversely, our previous research demonstrated PFC dysfunction during the Stroop color-word task in children with ASD, as measured via 24-channel NIRS, in comparison to typically developing children (35).

The heterogeneous findings from NIRS studies in children with ASD regarding prefrontal hemodynamic responses may be attributed to sample characteristics, particularly limited sample sizes and diagnostic imprecision. Furthermore, a limited number of NIRS studies have investigated prefrontal hemodynamic responses in adults with ASD. Consequently, in the present investigation, we assessed the hemodynamic responses of the PFC 24-Ch NIRS during a Stroop color-word task in a relatively large sample of 198 adult participants. Furthermore, all adults with ASD underwent a comprehensive Autism Spectrum Disorder Observation Schedule-Second Edition (ADOS-2) assessment (48), which is a commonly used diagnostic tool for ASD.

## 2 Methods

### 2.1 Participants

The study included 114 participants (85 males and 29 females) with ages ranging from 17 to 51 years, all of whom were diagnosed with ASD in accordance with the criteria outlined in the Diagnostic and Statistical Manual of Mental Disorders Fifth Edition (DSM-5) (1), with 84 typically developing control participants (46 males and 38 females) aged 18–44 years (**Table 1**). The individuals with ASD had no prior history of treatment for psychiatric disorders and had consulted an experienced psychiatrist at the Department of Psychiatry of the Nara Medical University. Individuals diagnosed with ASD underwent a standardized clinical assessment comprising a psychiatric evaluation, including the ADOS-2 (48), Mini International Neuropsychiatric Interview (Japanese version 5.0.0) (49), and a medical history examination by an experienced psychiatrist. Two experienced psychiatrists confirmed the ASD diagnosis according to DSM-5 (1). We also estimated each participant’s full-scale intelligence quotient (IQ) using the similarities and symbol search subsets of the Wechsler Adult Intelligence Scale-Third Edition, excluding individuals with IQ scores below 70.

**Table 1:** Participant characteristics.

Variable	ASD		Control		T value	P value
	(n = 114)		(n = 84)			
	Mean	SD	Mean	SD		
Sex (Male/female)^a^	85/29		46/38			**0.004**
Age (years)^b^	27.57	8.076	28.26	8.441	0.584	0.562
FIQ (WAIS-IV) ^b^	97.50	13.788	105.76	9.451	4.732	**<0.001**
AQ-J total score ^b^	30.57	7.216	16.67	7.429	-13.233	**<0.001**
AQ-J social skill ^b^	6.86	2.488	3.90	2.766	-7.874	**<0.001**
AQ-J attention switching ^b^	7.06	1.840	3.65	2.074	-12.196	**<0.001**
AQ-J local detail ^b^	4.90	1.858	3.74	2.123	-4.105	**<0.001**
AQ-J communication ^b^	6.57	2.562	2.33	2.147	-12.301	**<0.001**
AQ-J imagination ^b^	5.16	2.156	3.04	2.148	-6.857	**<0.001**
SCWC^b^	142.26	35.518	166.95	29.603	5.181	**<0.001**

ASD, autism spectrum disorder; FIQ(WAIS-IV), Full-scale IQ score of the Wechsler Adult Intelligence Scale, Fourth Edition; SD, standard deviation; AQ-J, Autism-Spectrum Quotient Japanese version; SCWC, Stroop color-word task number of correct answers.

a The χ^2^ test was used to test for group differences.

b Student’s t-test was used.The bold values mean p < 0.05.

We excluded patients with comorbid psychiatric disorders, such as ADHD, as defined by DSM-5^1^, neurological disorders, head injuries, serious medical conditions, or a history of substance abuse and dependence. The final sample included 114 participants with ASD who had no history of previous medications and the Japanese version of the autism-spectrum quotient Japanese (AQ-J) score of ≥26 (50–52). We recruited typically developing controls through local print advertisements. Typically developing participants underwent a standard clinical assessment comprising a psychiatric evaluation, standard diagnostic interviews, and a medical history examination conducted by two experienced psychiatrists. A psychologist evaluated the cognitive abilities of the participants utilizing the Wechsler Adult Intelligence Scale-Fourth Edition (WAIS-IV). Finally, 84 typically developing individuals without confirmed ASD and no current or history of psychiatric or neurological disorders were included in the study. The Institutional Review Board of Nara Medical University approved this study.

### 2.2 Assessment of ASD symptoms

The Autism Spectrum Quotient (AQ), developed by Baron-Cohen et al. based on the autism spectrum hypothesis, is a standardized assessment instrument designed to measure ASD traits and is widely utilized for ASD screening and research purposes (50). The Japanese version of the AQ, known as the AQ-J, has demonstrated satisfactory reliability and validity in screening young adults of normal intelligence for ASD, utilizing the same threshold values as the original AQ (50–52). The AQ-J is a self-administered instrument designed to quantify the degree of ASD characteristics or broad phenotype in adults with intelligence within the normal range. The Autism Quotient (AQ) comprises 50 items in total. The AQ is utilized not only for clinical screening but also to assess individual differences in autistic tendencies among typically developing adults; therefore, the AQ is advantageous for both diagnostic purposes and research endeavors (53).

### 2.3 Stroop color-word task

In this investigation, we employed a standard Stroop color-name task that incorporated word reading, incongruent color-name, and color-name conditions (54), and subsequently applied the previously established methodology (35). In this investigation, the word reading task served as the baseline condition, while the incongruent color-name task functioned as the activation condition. The color-name task, which is typically a component of the conventional Stroop color-word task, was omitted from this study. In the Stroop color-word task utilized in this study, each row contained 20 words, with a maximum of 5 rows, resulting in a total of 100 words per page. The initial page comprised the chromatic terms RED, GREEN, and BLUE printed in black ink. On the second page, the words RED, GREEN, and BLUE were printed in red, green, or blue ink, respectively, with the color of the ink not corresponding to the semantic meaning of the word. The lexical items on both pages were distributed randomly, and identical elements were not positioned within the same row.

The procedure was conducted by a qualified psychologist, who initially provided the participants with the following instructions: “For this task, you will first read the words on the initial page, and subsequently, on the second page, you will respond as promptly as possible by indicating your selections through word coloration.” Upon the verbal cue “start,” read the words in the columns from the top left, and respond as promptly as possible by indicating the words or their respective colors. After completing the words in the first column, proceed to the subsequent column. The participant read out the content on the second page for 45 seconds, then proceeded to turn the page over. This process is repeated three times on the second page. Subsequently, the first page is presented again.

**Figure 1:**
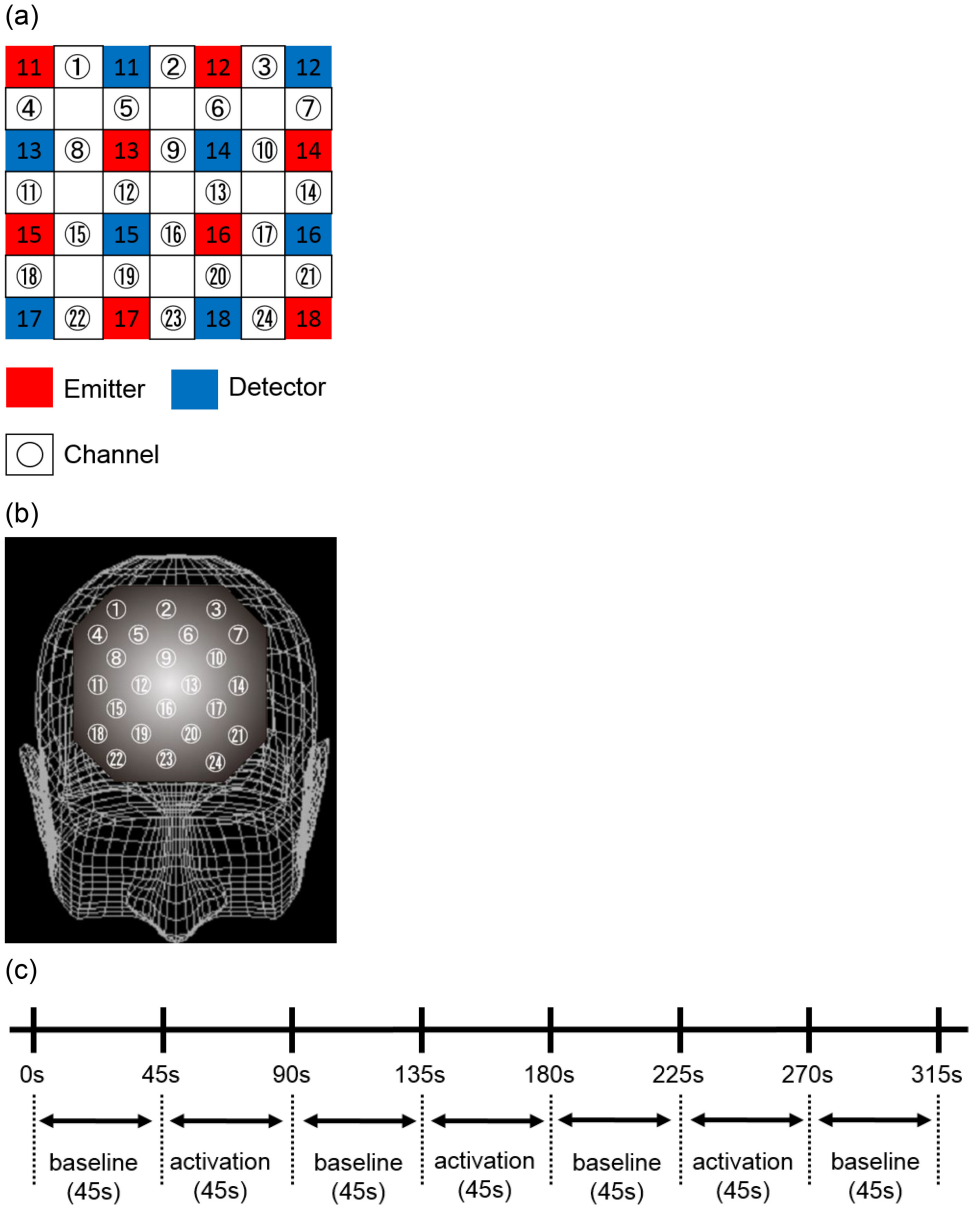
Location of the 24 channels in the near-infrared spectroscopy instrument. **(A)** Placement of emitters and detectors and definition of each channel. **(B)** Corresponding anatomical sites for each channel. **(C)** Data analysis protocols and procedures for each task. The baseline and activation tasks for all Stroop color-word tasks performed were 45 seconds each. These tasks were repeated three times, followed by the baseline task.

The complete protocol of the Stroop Color Word Task comprised a 45-second task (p1), three 45-second tasks (p2), and a subsequent 45-second p1 task (**Figure 1A**). The 45-second p1 task (word reading task) served as the baseline task, while the p2 task (incongruent color naming task) functioned as the activation task. The number of correct responses in each trial was operationalized as follows: the number of correct responses in the first Stroop Color Word Task (SCWC) (21).

### 2.4 NIRS measurements

NIRS is a spectroscopic method that utilizes the near-infrared region (780nm to 2500nm) of the electromagnetic spectrum. NIRS quantifies the components by irradiating the target with near-infrared light and measuring the change in absorbance. Through the measurement of [oxy-Hb] and deoxygenated hemoglobin [deoxy-Hb], the hemodynamic responses (cortical activity) of the cerebral cortex can be evaluated non-invasively. The hemodynamic response is characterized by an increase in [oxy-Hb] and a decrease in [deoxy-Hb] and is based on the modified Lambert-Beer law, which monitors the absorption of near-infrared light by ([oxy-Hb] and [deoxy-Hb] at two distinct wavelengths (55, 56). The direction of change in [deoxy-Hb] is determined by the degree of oxygenation and volume change of venous blood, and studies utilizing rodent models have demonstrated that [oxy-Hb] serves as a more sensitive indicator of regional cerebral blood flow compared to alternative methods (57). In this investigation, as in the preceding study, we focused on the change in[oxy-Hb] to evaluate hemodynamic responses.

For the measurement of [oxy-Hb], a 24-channel NIRS device (Hitachi ETG-4000, Hitachi Medical, Tokyo) that measures the absorption of near-infrared light at two wavelengths (760 nm and 840 nm) was utilized, and [oxy-Hb] was calculated using the aforementioned method. The optical topography (NIRS) device positioned a light source and a light-receiving sensor 3 cm apart on the head and measured the change in [oxy-Hb] concentration 2–3 cm below the scalp, corresponding to the depth of the cerebral cortex.

NIRS measurements were conducted with participants in a seated, relaxed position. To minimize motion artifacts, participants were carefully monitored and instructed to avoid bodily movements that could potentially induce artifacts, such as neck movement, clenching, and blinking. The NIRS probe was positioned over the prefrontal cortex, and the relative [oxy-Hb] concentration change was measured at 24 measurement points within an 8 × 8 cm area (**Figure 1B**). The middle row of probes was aligned with Fpz, and the bottom row with the Fp1-Fp2 line, in accordance with the international 10-20 system utilized in EEG measurements. The positions of the probes and measurement points were verified by superimposing them on a 3D reconstructed MRI scan of the cerebral cortex of a representative control subject (**Figure 1C**). A virtual registration method (58) was used to estimate the cortical area for each channel employed to estimate the cortical area for each channel.

This probe configuration facilitated the measurement of changes in [oxy-Hb] concentration within the cortical surface areas of the frontopolar and anterior PFC (Brodmann area [BA] 10), dorsolateral PFC (BA 9 and 46), and ventrolateral PFC (BA 44 and 45). Near-infrared light absorption was measured in real time with a temporal resolution of 0.1 s, and the data were analyzed utilizing the “integration mode”. The pre-task baseline was defined as the mean activity for 10 s immediately preceding the task, and the post-task baseline was defined as the mean activity for 25 s immediately following the task. Linear fitting was subsequently performed on the data between these two baselines (21, 27). A moving average method was employed to eliminate short-term movement artifacts (moving average window was 5 s).

**Figure 2:**
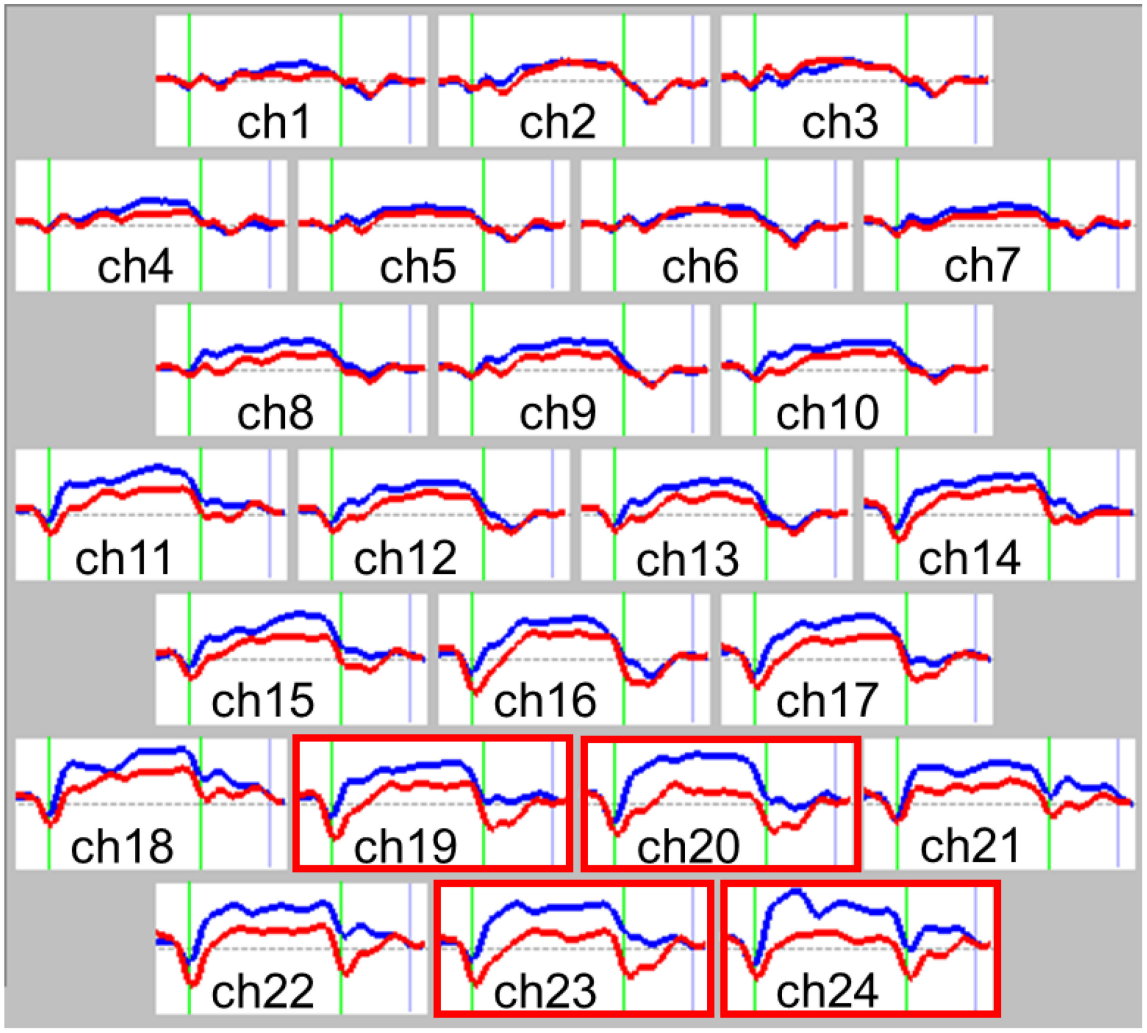
Grand average waveforms of changes in oxygenated hemoglobin (oxy-Hb) concentration change during the Stroop color-word task. Comparison of near-infrared spectroscopy (NIRS) waveforms (changes in oxy-Hb concentration) between the autism spectrum disorder (ASD) and control groups during the Stroop color-word task for each of the 24 recording channels. Red lines represent the autism spectrum disorder group, while blue lines represent the control group. The task was performed in the interval represented by green lines; the first green line indicates the beginning of the task and the second indicates the end. Significant region is shown in the red frame (Ch 20, 23, 24). Group differences were tested by multiple comparison test using the Bonferroni correction. P<0.05. Ch, channel.

NIRS results were analyzed by an examiner blinded to the participants’ diagnoses. Relative [oxy-Hb] was defined as the difference between [oxy-Hb] at baseline and during activation (**Figure 2**). The temporal profile of [oxy-Hb] change was evaluated from the initiation of the activation task until the [oxy-Hb] waveform returned to baseline. These values were averaged from three repeated trials to mitigate the effects of potential incidental changes and participant fatigue, consistent with previously described methodologies (21, 22, 27).

### 2.5 Statistical analyses

We used the χ2 test to examine group differences for categorical variables (male and female). For normally distributed clinical variables, we used Student’s t-test. We conducted analyses of covariance to control for FIQ scores and sex, with the region of interest (1–24 ch) as within-subject factors and group (ASD, control) as a between-subjects factor for the averaged [oxy-Hb] and [deoxy-Hb] concentration data. We applied Bonferroni correction to adjust for multiple comparisons. Additionally, we conducted multiple linear stepwise regression analyses to determine the independent factors influencing the NIRS components. The independent variables utilized were the total score and five sub-scales of the AQ-J (social skills, attention switching, local details, communication, and imagination) for both the control and ASD groups, and ADOS-2 score for the ASD groups exclusively. We set statistical significance at p < 0.05. All statistical analyses were conducted using IBM SPSS Statistics version 28 (IBM Corp., Armonk, NY, USA).

## 3 Results

### 3.1 Demographic and clinical data

Demographic characteristics are presented in **Table 1**. The participant groups differed in terms of sex (X^2^ = 8.469, *p* = 0.004) and FIQ score (*t*_19 6_ =4.732, *p <*0.001). However, there was no difference in average age (*t*_196_ = 0.584, *p* = 0.560). The ASD group had significantly higher scores on five sub-scales of the AQ-J (social skills, attention switching, local details, communication, and imagination) and the total score (*t*_196_ = -13.233, *p <*0.001; *t*_196_ = -7.874, *p <*0.001; *t*_196_ = -12.196, *p <*0.001; *t*_196_ = -4.105, *p <*0.001; *t*_196_ = -12.301, *p <*0.001; *t*_196_ = -6.857, *p <*0.001 respectively). There were significant differences in SCWC scores between the two groups (*t*_196_ = 5.181, *p <*0.001).

### 3.2 NIRS data during the Stroop color-word task

The grand average waveforms of [oxy-Hb] changes during the Stroop color word task in both groups are illustrated in **Figure 2**. We found significant between-group differences in the mean [oxy-Hb] changes during the task period in four channels (Ch-19: *F*_1.4537_ = 10.241, *p* = 0.033; Ch-20: *F*_1.4537_ = 20.370, *p <*0.001; Ch-23: *F*_1.4537_ = 19.134, *p <*0.001; Ch-24: *F*_1.4537_ = 22.350, *p <*0.001, Bonferroni-corrected) located near the orbitofrontal cortex and frontal pole (**Table 2**).

**Table 2:** Difference of mean oxygenated hemoglobin (oxy-Hb) measurements between task and post-task periods in 24 channels.

	ASD (mMmm)		Control (mMmm)		F-value	*p*-value
	Mean	SD	Mean	SD		
Ch1	-0.0008	0.0712	0.0026	0.0652	0.013	1.000
Ch2	0.0024	0.0820	0.0074	0.0675	0.003	1.000
Ch3	0.0185	0.0674	0.0090	0.0648	1.083	1.000
Ch4	0.0115	0.0687	0.0168	0.0578	0.004	1.000
Ch5	0.0054	0.0683	0.0115	0.0510	0.021	1.000
Ch6	0.0076	0.0690	0.0117	0.0594	0.001	1.000
Ch7	0.0062	0.0677	0.0120	0.0766	0.014	1.000
Ch8	0.0084	0.0733	0.0290	0.0613	1.547	1.000
Ch9	0.0089	0.0723	0.0234	0.0711	0.005	1.000
Ch10	0.0094	0.0666	0.0252	0.1000	0.788	1.000
Ch11	0.0227	0.0911	0.0571	0.0840	5.202	0.552
Ch12	0.0082	0.0895	0.0296	0.0802	1.935	1.000
Ch13	0.0052	0.0676	0.0275	0.0813	1.681	1.000
Ch14	0.0174	0.1004	0.0445	0.1123	2.974	1.000
Ch15	0.0163	0.0923	0.0472	0.0814	4.086	1.000
Ch16	0.0083	0.1071	0.0362	0.0942	3.289	1.000
Ch17	0.0090	0.0980	0.0426	0.0993	4.950	0.624
Ch18	0.0396	0.1137	0.0752	0.1239	5.710	0.408
Ch19	0.0024	0.1029	0.0483	0.1130	10.241	**0.033**
Ch20	-0.0067	0.0872	0.0567	0.1341	20.370	**<0.001**
Ch21	0.0175	0.0954	0.0604	0.1176	8.506	0.085
Ch22	0.0122	0.1279	0.0636	0.1064	12.881	0.008
Ch23	-0.0028	0.1058	0.0587	0.1230	19.134	**<0.001**
Ch24	0.0008	0.0968	0.0673	0.1240	22.350	**<0.001**

Group differences tested with Analyses of Covariance (ANCOVA) to control FIQ and Sex.The bold values mean p < 0.05.

### 3.3 Multiple regression analyses in ASD and control

Multiple regression analyses in both the ASD and control groups revealed that the AQ-J total score significantly contributed to mean [oxy-Hb] changes during the Stroop color-word task at Ch-20 (β = -0.196, p = 0.043). Furthermore, AQ-J attention switching significantly contributed to mean [oxy-Hb] changes during the Stroop color-word task at Ch-20 (β = -0.255, p = 0.009) (**Table 3**). Conversely, the ADOS-2 score in the ASD group alone did not significantly contribute to mean [oxy-Hb] changes during the Stroop color-word task at any channel.

**Table 3:** Multiple regression analysis and 24 channels.

Dependent variable and covariate	B	SE	β	*t*-value	*p*-value	R^2^	Adjusted R^2^	*p*-value
AQ-J total						0.155	0.132	**0.001**
Ch20	-17.187	8.453	-0.196	-2.033	**0.043**			
Ch23	0.422	9.002	0.005	0.047	0.963			
Ch24	-5.989	9.367	-0.068	-0.639	0.523			
AQ-J social skill						0.053	0.028	0.144
AQ-J attention switching						0.158	0.136	**0.003**
Ch20	-5.712	2.152	-0.255	-2.654	**0.009**			
Ch23	-0.183	2.292	-0.008	-0.080	0.936			
Ch24	-0.219	2.385	-0.010	-0.092	0.927			
AQ-J local detail						0.054	0.029	0.321
AQ-J communication						0.120	0.096	**0.002**
Ch20	-2.633	2.756	-0.094	-0.955	0.341			
Ch23	-1.503	2.935	-0.056	-0.512	0.609			
Ch24	-1.218	3.054	-0.043	-0.399	0.690			
AQ-J imagination						0.122	0.099	**0.001**
Ch20	-2.199	2.062	-0.105	-1.066	0.288			
Ch23	-1.663	2.196	-0.082	-0.757	0.450			
Ch24	-0.742	2.285	-0.035	-0.325	0.746			

AQ-J, Autism-Spectrum Quotient Japanese version; Ch, channel; SE, standard error.The bold values mean p < 0.05.

## 4 Discussion

To the best of our knowledge, no previous studies have examined the broader prefrontal hemodynamic response in adults with ASD measured using 24-Ch NIRS during the Stroop color-word task. The results of the present study revealed that the [oxy-Hb] changes in 114 adults with ASD during the Stroop color-word task were significantly smaller than those in the PFC of 84 typically developing controls, particularly in the dorsolateral PFC (Ch-19, -20, -23, and -24) (**Table 2**).

The current findings corroborate our hypothesis and align with the proposed prefrontal cortex (PFC) dysfunction in ASD, as identified through various functional neuroimaging techniques, including fMRI and single-photon emission computed tomography (SPECT). SPECT studies on autism spectrum disorders have demonstrated a reduction in regional cerebral blood flow across various brain regions, including the insula, superior temporal gyrus, prefrontal cortex, cingulate gyrus, temporal lobe, and parietal lobe (59–61). A systematic review and meta-analysis of fMRI studies of autism spectrum disorders has indicated the PFC dysfunction (62). We previously measured hemodynamic responses in the PFC during a Stroop color-word task using 24-Ch NIRS in 12 drug-naive male children with ASD aged 7–15 years according to the DSM-5 criteria, and 12-year-old- and FIQ-matched typically developing control males. We found that oxyhemoglobin changes during the Stroop color-word task in the ASD group were significantly smaller than those in the control group in Ch-12 and -13, located over the bilateral dorsolateral PFC (35). In the present study, our findings indicate that at Ch-19, -20, -23, and -24, adults with ASD exhibited significantly smaller [oxy-Hb] changes compared to typically developing controls (**Table 2**). These channels were also localized in the orbitofrontal cortex and frontal pole, which differs from the findings of our previous study in children. The discrepancies between the results of these two studies may simply be due to differences in sample size between these studies. However, it is undeniable that the dysfunction of the PFC in individuals with ASD may be affected by age, and we need to give sufficient consideration to the longitudinal course of neuroanatomy in the central nervous system.

The developmental trajectory of human brain regions exhibits variability. The ontogenetic curve of areas responsible for higher cognitive functions, particularly executive functions, which are predominantly associated with the PFC, initiates later and extends for a longer duration compared to other cerebral regions (63–65). PFC plays a crucial role in higher cognitive functions, and certain developmental abnormalities occurring during the period from adolescence to early adulthood may be associated with the onset of psychiatric disorders, including ASD and schizophrenia (66, 67). This distinctive developmental process in the PFC correlates with the maturation of higher cognitive function during adolescence and early adulthood (63). For instance, an fMRI study utilizing an inhibition task related to self-regulation demonstrated that the blood oxygen level-dependent signal increased with age, from 9 to 11 years, but subsequently decreased progressively into adulthood, following the same inverse U-shaped trajectory as developmental changes in prefrontal gray matter volume (68, 69). Comprehension of this process may facilitate a thorough understanding of the discrepancies in results between the two studies.

The [oxy-Hb] changes at Ch-20 exhibited significant correlation with the AQ-J total and attention switching scores, which represent a symptom cluster of the AQ-J (**Table 3**). Ch-20 is anatomically localized to the PFC, specifically in proximity to the frontal pole, a cortical region situated at the anterior margin of the PFC. This area is characterized as the most tardily myelinated cortical region (70). The precise function of the frontal pole in higher cognitive processes remains to be elucidated. Nevertheless, extant research has indicated that the frontal pole is implicated in the concurrent processing of multiple tasks involving complex attentional functions (71, 72) and may play a significant role in attentional processes, including alternating attention. Individuals with ASD exhibit impaired attentional function, a cognitive deficit that suggests that diminished activity in the PFC, particularly in the frontal pole, during the Stroop color-word task in patients with ASD is associated with compromised attentional function.

Previous studies reported reduced PFC activity in patients with ASD during cognitive control tasks involving inhibition (73), attention (74, 75), and working memory (76, 77). In the present study, we used the Stroop color-word task because the PFC is one of the regions most strongly associated with Stroop interference (78). We previously reported PFC dysfunction during the Stroop color-word task in individuals with pediatric ASD, as measured by NIRS, compared to typically developing children (35). Our study incorporated a larger sample size than previous NIRS studies on ASD, indicating that NIRS measurements in adults with ASD could be a valuable instrument to aid in the clinical autism diagnostic process. The validity of our data was further strengthened by the inclusion criterion of an AQ-J (50–52) score of ≥26 to minimize diagnostic uncertainty in the ASD group.

This study presents several potential limitations. First, NIRS exhibits certain disadvantages compared to other functional neuroimaging techniques (79): for instance, NIRS only enables the measurement of Hb concentration changes as relative values. To address these potential issues, we employed the Stroop color-word task with a distinct baseline task. Furthermore, we measured Hb concentration changes between the activation and baseline tasks and conducted the task three times to average the potential effects of incidental changes and mitigate participant fatigue. The grand average waveforms of [oxy-Hb] concentration changes in the ASD group did not indicate a decrease in regional cerebral blood flow during the activation task or a difference in blood flow between baseline and activation tasks. Second, the spatial resolution for detecting hemodynamic responses from the scalp surface using NIRS is lower than that of fMRI, SPECT, and PET. However, abnormal prefrontal hemodynamic responses in individuals with ASD can be detected using NIRS. Third, several studies have shown that superficial hemodynamic responses such as skin blood flow can affect prefrontal NIRS hemoglobin signals (80, 81). Consequently, the present findings may have been influenced by skin blood flow. However, Sato et al. (82) conducted simultaneous NIRS, fMRI, and laser Doppler flowmeter measurements to determine whether prefrontal NIRS hemoglobin signals reflected cortical activity rather than superficial effects. They concluded that NIRS could be used to measure the hemodynamic signals originating from PFC activation. Fourth, the study included patients who were not drug naïve. The possibility that psychotropic drugs affect PFC function cannot be eliminated. Thus, our findings are not generalizable to all adults with ASD. Nevertheless, our current conclusion, is that abnormal prefrontal hemodynamic responses in adults with ASD are valuable for extending current knowledge. Further research is required to confirm these findings.

## 5 Conclusion

To the best of our knowledge, no previous studies have examined the broader prefrontal hemodynamic response in adults with ASD measured using 24-Ch NIRS during Stroop color-word task. We observed that the changes in [oxy-Hb] concentrations in the PFC were significantly lower in adults with ASD compared to typically developing controls. Furthermore, the changes in [oxy-Hb] concentrations at Ch-20 showed a significant correlation with the AQ-J total score and the attention switching score. These results demonstrate that adults with ASD may exhibit PFC dysfunction, and suggest that the reduced activity of the PFC, especially the frontal pole, in the Stroop color-word task is related to reduced attention function. Given that the multichannel NIRS system facilitates non-invasive functional mapping of the cerebral cortex and requires a substantially shorter measurement time (approximately 5 min) than other functional brain imaging methodologies, it may serve as an effective tool for ASD diagnosis.

## Data Availability

The datasets presented in this study can be found in online repositories. The names of the repository/repositories and accession number(s) can be found in the article/supplementary material.
